# Pituitary apoplexy: an update on clinical and imaging features

**DOI:** 10.1007/s13244-014-0362-0

**Published:** 2014-10-16

**Authors:** Alessandro Boellis, Alberto di Napoli, Andrea Romano, Alessandro Bozzao

**Affiliations:** 1 Neuroradiology NESMOS Department, University of Rome “La Sapienza” and Azienda Ospedaliera Sant’Andrea, Via di Grottarossa, 1035/1039, Rome, Italy; 2 Fondazione San Raffaele, Ceglie, Messapica, Italy

**Keywords:** Pituitary apoplexy, Pituitary adenoma, MRI, Pituitary haemorrhage, Macroadenoma

## Abstract

Pituitary apoplexy (PA) is a rare and potentially fatal clinical condition presenting acute headache, vomiting, visual impairment, ophthalmoplegia, altered mental state and possible panhypopituitarism. It mostly occurs in patients with haemorrhagic infarction of the pituitary gland due to a pre-existing macroadenoma. Although there are pathological and physiological conditions that may share similar imaging characteristics, both clinical and imaging features can guide the radiologist towards the correct diagnosis, especially using magnetic resonance imaging (MRI). In this review, we will describe the main clinical and epidemiological features of PA, illustrating CT and MRI findings and discussing the role of imaging in the differential diagnosis, prognosis and follow-up.

*Teaching points*

• *Headache, ophtalmoplegia and visual impairment are frequent symptoms of pituitary apoplexy.*

• *CT is often the first imaging tool in PA, showing areas of hyperdensity within the sellar region.*

• *MRI could confirm haemorrhage within the pituitary gland and compression on the optic chiasm.*

• *Frequent simulating conditions are aneurysms, Rathke cleft cysts, craniopharingioma and mucocele.*

• *The role of imaging is still debated and needs more studies.*

## Introduction

Pituitary apoplexy (PA) was first described by Pearce Bailey in 1898 [1] and recognised as a defined clinical syndrome by Brougham et al. in 1950 [2]. It is a severe and potentially fatal medical condition characterised by the variable association of headache, vomiting, visual impairment, ophthalmoplegia, altered mental state and panhypopituitarism [2–5]. The syndrome is often related to haemorrhagic infarction of the pituitary gland, usually in a condition of a pre-existing macroadenoma [6–8] occurring in 1.6 to 2.8 % of patients with this neoplasm. In a consistent number of patients with PA, this clinical syndrome represents the first sign of a previously undetected adenoma. Few cases of apoplexy have been reported in patients without a previous pituitary pathology [9, 10] such as Sheehan syndrome, a condition characterised by pituitary infraction occurring in post- or peripartum females with hypovolaemia [11–13].

The diagnosis of PA can be made only when haemorrhagic infarction of the pituitary gland leads to the previously described clinical syndrome; the use of the term PA should be avoided in asymptomatic patients. In fact, huge haemorrhages can be a common finding in patients with macroadenomas without PA symptoms [14, 15].

Many clinical series of patients with PA have been reported, while its imaging features are less frequently described. Because of the potential severe prognosis of PA, radiologists should be aware of its imaging characteristics on both computed tomography (CT) and magnetic resonance imaging (MRI), being able to recognise the main conditions that may mimic PA, from a clinical and radiological perspective.

## Definition, clinical features and pathogenesis

Based on the above-mentioned clinical definition, PA is a rare event and occurs in 1.6 to 2.8 % of patients with macroadenomas; it is more frequent in males, with a gender ratio of 2:1 and a mean age of onset of 57 years [5, 16–18]. About 60 % of patients with PA may not be aware of having a pituitary adenoma before the acute event.

Pituitary apoplexy has a wide spectrum of clinical manifestations; based on many reports, the most common symptom is headache, with an incidence of 90–97 % [2–5, 19]. This is often described as a sudden, severe head pain, frequently retro-orbital in location [20], that includes PA into the differential diagnosis of a condition known as “thunderclap headache”. This comprehends important and more frequent diseases such as subarachnoid haemorrhage, cerebral venous sinus thrombosis or cervical artery dissection [21].

The second most frequent symptom is the visual deficit, ranging from 50 to 82 % of cases [5, 13, 20–25], followed by nausea, vomiting, ocular palsy and meningism (25–50 %).

Many authors described that PA is strictly associated with a sudden enlargement of a pre-existing pituitary adenoma, resulting in a disturbance of the residual pituitary function and local mass effect on surrounding structures or, less frequently, in a sudden increase of intracranial pressure [5, 16, 26–28]. Meningeal irritation occurs if blood gains access to the suprasellar cisterns through a dilated diaphragm sellae [5].

Many known factors predispose patients to symptomatic infarction of the pituitary, amongst which the most important are hypertension [10, 20], diabetes mellitus [29], pituitary function dynamic tests [30, 31], administration of anticoagulants, bromocriptine, oestrogens [32–34] and radiotherapy [35]. The pathogenesis underlying pituitary apoplexy is still unknown. A possible mechanism can be the subacute, excessive growth of the pre-existing adenoma, which outgrows its blood supply with eventual ischaemic necrosis followed by haemorrhage [2, 35]. Indeed, pathologic and dynamic imaging studies [36, 37] have shown that macroadenomas, as well as microadenomas, are less vascularised than the pituitary gland, so that a relatively fast and sizable growth can exceed this low blood supply. However, this theory does not explain the onset of PA in patients with small adenomas or with a healthy pituitary. Another hypothesis is that tumour compression of the infundibulum and superior pituitary arteries may cause infarction of the normal pituitary gland; however, ischaemia of the tumour mass itself is less probable in this case because the vessels supplying the adenoma are attributable to the inferior pituitary circulation [38]. Therefore, pituitary tumours probably suffer from an intrinsic vasculopathy that can lead to spontaneous infarction and haemorrhage [4, 22, 23, 39].

## Imaging findings

### Computed tomography

**Fig. 1:**
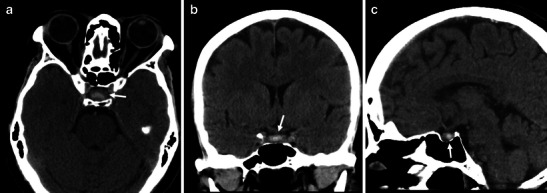
CT appearance of pituitary apoplexy. Arrows on axial (**a**), coronal (**b**) and sagittal (**c**) reconstructions indicate an intrasellar lesion, dis-homogeneously hyperdense, due to the presence of haemorrhagic components. The utility of reformatted images in the evaluation of the intrasellar hyperdensity is evident

Most of the patients with symptoms related to PA will undergo computed tomography (CT) in an emergency setting. It is likely that, in most of them, the clinical suspicion might something other than PA. CT is effective in visualising pituitary expansive lesions leading to sellar enlargement (up to 94 % of cases) [5, 20, 25]. Because of the haemorrhagic component in most of PAs, CT will show patchy or confluent areas of hyperdensity within a pituitary lesion (Fig. 1) [5, 14, 20, 25]. Although a hyperdense lesion inside the sella turcica tends to represent PA in the proper clinical setting, diseases other than PA may lead to similar radiological pictures. The most common hyperdense lesions in the pituitary region are aneurysms, meningiomas, Rathke cleft cysts, germinomas and lymphoma. They can be easily differentiated from blood using MRI. After administration of contrast medium, a rim of enhancement may be the sign of pituitary apoplexy [40]. The real accuracy of CT in the diagnosis of PA is not fully described, being dependent on the time of imaging from onset of symptoms and on technical characteristics of CT itself. Some authors demonstrated a low sensitivity of CT in detecting pituitary tumour haemorrhage; this ranges from 21 % [20] to 46 % [16]. A possible explanation for these wide ranges is related to blood degradation, leading to a decreased density, in the days following the acute onset. For this reason, making the distinction between cystic or degenerative changes inside a pituitary neoplasm and a subacute haemorrhage may be impossible [14]. Based of the above-mentioned data, it is important to remember that the CT examination may be normal, underestimated or non-pathognomonic, especially in those rare patients with non-haemorrhagic PA or without a pre-existing pituitary adenoma. Therefore, although CT can exclude other diseases (i.e. subarachnoid haemorrhage), an MR examination can give more details about a suspected PA [14, 20].

### Magnetic resonance imaging

Magnetic resonance imaging (MRI) is the most important tool in the diagnosis of PA, being able to identify the presence of an adenoma and its haemorrhagic degeneration. MRI is definitely superior to CT in the diagnosis of PA with a sensitivity ranging from 88 % to 90 % [18, 41]. Recently, Semple et al. demonstrated that MRI features of PA correlate with the pathologic reports and operative findings and that PA features can be accurately predicted from MR imaging [14].

**Fig. 2:**
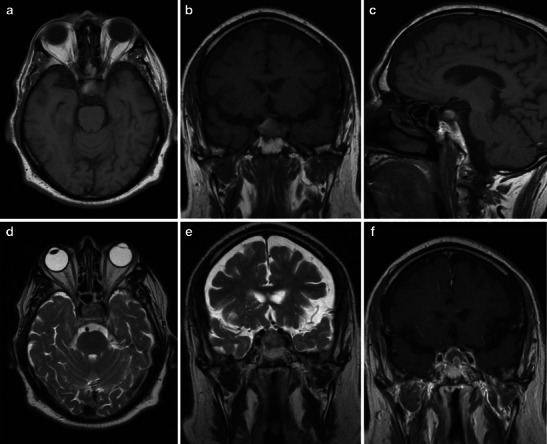
MRI performed in a patient with acute headache, mydriasis and visual impairment shows acute/early subacute phase haemorrhagic infarction within a pituitary macroadenoma. The intrasellar mass appears slightly hyperintense on unenhanced T1WI (axial, **a**; coronal, **b**; sagittal, **c**) with areas of hypointense signal on T2WI (axial, **d**; coronal, **e**) because of the presence of deoxyhaemoglobin. Coronal T1 contrast-enanched images (**f**) show left cavernous sinus involvement, probably leading to visual symptoms

**Fig. 3:**
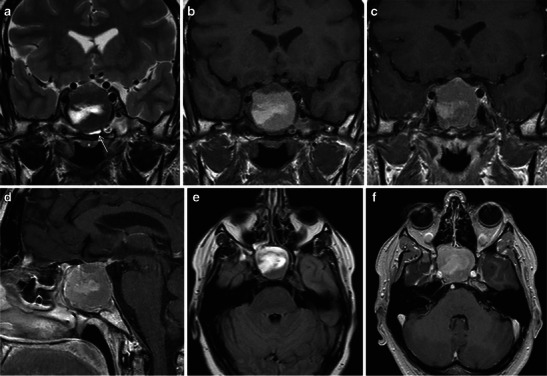
Large pituitary macroadenoma with MRI signs of subacute haemorrhage. In this phase, haemorrhage appears hyperintense on both T1WI (**b**) and T2WI (**a**). In the T2WI (**a**, **e**), thickening of the sphenoid mucosa (arrow) is evident as well. This sign is highly specific for pituitary apoplexy. On contrast-enhanced MRI (**c**, **d**, **f**) the residual pituitary gland enhances (arrow in **c** compared to **b**). Note the optic pathway compression, more evident on T2WI

**Fig. 4:**
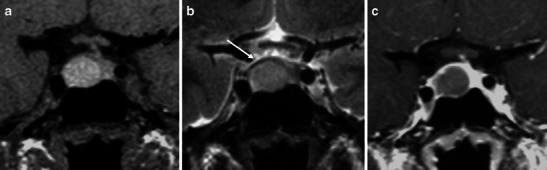
The figure shows an oval-shaped, haemorrhagic lesion within a pituitary adenoma, homogeneously hyperintense on both T1- and T2-weighted images (WI) (**a**), with a hypointense rim on T2WI (**b**, white arrow). Contrast-enhanced T1WI (**c**) shows strong, homogeneous enhancement of the pituitary gland and stalk, both displaced by the tumour

It is well known that the signal of blood clots in MRI changes over time. In the acute phase (0–7 days), deoxyhaemoglobin leads to shortening of the T2 relaxation time due to the susceptibility effect and the MRI signal is hypointense on T2-weighted imaging (T2W1) with isointensity or slight hypointensity on T1-weighted imaging (T1WI) (Fig. 2). In the subacute phase (7–21 days), methaemoglobin shortens the T1 relaxation time and the haemorrhage will appear hyperintense on T1WI as well as on T2WI (Figs. 3 and 4). In the chronic phase (>21 days), macrophages digest the clot and the presence of haemosiderin and ferritine causes a strong hypointensity on both T1WI and T2WI [42–44].

First, Poitin et al. and, more recently, Dubuisson et al. described the MRI features in a group of patients with PA [5, 45]. MRI predominantly showed an intra- and suprasellar expanding mass with different signal intensities on T1WI and T2WI, depending on the presence of haemorrhage and on its stage, as described above. In most cases, MRI performed during the acute phase of the clinical syndrome shows areas of hyperintensity on T1W1, generally more pronounced in the peripheral area of the lesions [45]. After IV gadolinium administration, a slight and inhomogeneous contrast enhancement is evident (Figs. 3c, f, 4) but often is difficult to differentiate from normal residual pituitary gland [5, 14, 45, 46]. T2WI showed areas of mixed high and low signal intensity, with the presence of a possible thin peripheral ring of marked hypointensity, representing haemosiderin and ferritin deposition. The latter must be considered an unusual and unexpected finding, since pituitary tumours lack a blood-brain barrier, and the accumulation of macrophages containing haemosiderin normally does not occur [45]. T2WIs are even useful in the evaluation of potential compressions of the optic chiasm and hypothalamus by the tumour [40].

Many authors [5, 14, 45] described the possibility of a fluid debris level within the mass. Poitin et al. made a pathological-radiological correlation in their patients with this finding, showing that the upper fluid (hyperintense on T1W1) contains free extracellular meta-Hb and the lower layer (iso-hypointense on T1WI) corresponds to a sediment of red blood remnants [45]. This finding is suggestive of late subacute haemorrhage and is considered a specific sign of pituitary apoplexy [45–47].

Another important MRI finding that is highly indicative of PA is the thickening of sphenoid sinus mucosa (Fig. 3a) [14, 46, 48]. This condition may be related to venous engorgement in this region, a reversible condition on follow-up studies [14]. Thus, this is considered a specific finding of the acute phase of haemorrhage.

Less is known about the MRI appearance of pituitary apoplexy without haemorrhagic infarction. Ostrov et al. found that this condition may appear as low signal intensity on both T1WI and T2WI, without contrast enhancement inside the lesion but with a peripheral rim of enhancement [49]. It has been reported that diffusion-weighted images may help to visualise areas of infarction within a non-haemorrhagic pituitary lesion [15] but obtaining good DWI images in the pituitary region is challenging because of susceptibility artefacts.

The use of T2*-weighted gradient-echo (GE) MR imaging is currently the most sensitive neuroimaging technique for the identification of brain haemorrhage. T2*-weighted images are highly sensitive to the paramagnetic effects of deoxy-HB and meta-HB and show bleeding products and haemosiderin deposits as areas of strongly hypointense signal. Tosaka et al. recently demonstrated that this statement could be applied even to PA [50]. GE MR can depict acute and chronic haemorrhages not seen with conventional SE techniques. These can appear as a hypointense rim, mass, spot or diffused area of lower signal intensity. The latter, diffused, is related to pituitary adenomas without a history of headache or clinical PA.

### Differential diagnosis

MRI is a useful tool in the differential diagnosis of pituitary apoplexy. Many conditions can present with hyperintensity  on T1WI in the sellar region, the most frequent MRI feature of PA [46]. Thus, it is important to use advanced MRI techniques such as T2*GE together with a careful search for specific radiological findings, such as fluid levels or thickening of the sphenoid mucosa.

The posterior lobe of the pituitary gland usually appears as a hyperintense spot on T1WI because of storage of vasopressin [51–53]; this is a normal finding and should not be confused with a haemorrhage within the gland.

In the case of anterior pituitary lobe hyperactivity, the gland may be hyperintense [54, 55]. In these cases, haemorrhage can be easily excluded with the evaluation of T2WI and T2*WI.

**Fig. 5:**
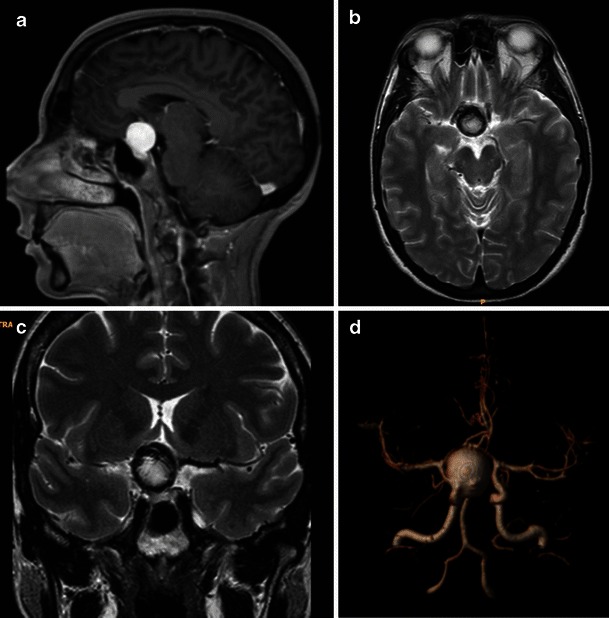
Aneurysm of the carotid siphon as shown on contrast-enhanced T1WI (**a**, arrows). Axial and coronal T2WI (**b**, **c**) show the presence of a hyperintense lesion with a peripheral hypointense rim. Contrast-enhanced MR angiography with 3D reconstruction (**d**) clearly shows the exact nature of the lesion

Another condition to consider for the differential diagnosis is the presence of an aneurysm (Fig. 5). Aneurysms arising from the carotid siphon or from the anterior communicating artery usually appear as round lesions, hypointense on T2WI because of flow void. Partially thrombosed aneurysm in the sellar region can show high signal intensity on T1WI [46]. Thus, an imaging study should be completed with MR angiography, which is best with a contrast-enhanced technique, to exclude this pathology [25].

**Fig. 6:**
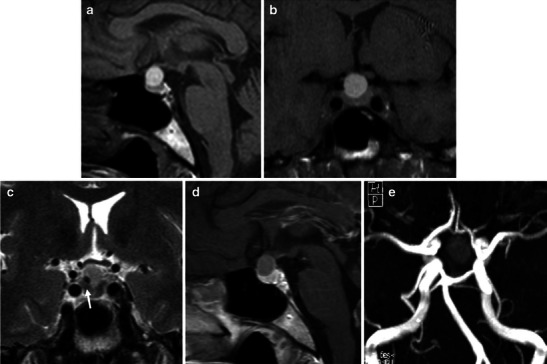
Suprasellar RCC appears hyperintense on T1WI (**a**, **b**) without contrast enhancement (**d**). On coronal T2WI (**c**), an arrow indicates an intracystic hypointense nodule, a typical sign of RCC, related to proteinaceous concretion. MR angiography (**e**) can be useful to rule out the possibility of an aneurysm, but the cyst will appear mildly hyperintense on MIP reconstruction

Rathke cleft cysts (RCCs) occur with T1 hyperintensity because of the variable protein content [56] (Fig. 6). RCC, which can be either intra- or suprasellar, is usually located along the midline and does not present a fluid debris level [40]. A hypointense intracystic nodule on T2WI, related to concretion of proteinaceous material within the RCC, is typical (Fig. 6c). The variable MRI appearance of RCC may cause difficulties in the differential diagnosis with PA. Moreover, a large suprasellar RCC may mimic PA even clinically, with acute onset of headache and visual deficit, as described by the recent work of Binning et al. [57].

Craniopharingioma is another pathology that can be difficult to distinguish from PA. It is usually an intra-/suprasellar lesion with variable solid, cystic and calcified components [40, 58]. The cystic component may contain a proteinaceous fluid, appearing hyperintense on T1WI, and mimicing haemorrhagic degeneration. Careful evaluation of T2WI together with the acquisition of a CT scan to rule out calcific components can help to obtain the correct diagnosis [40].

Other conditions that can simulate PA on imaging are lipoma, dermoid cysts and postoperative changes. Lipoma and dermoid cysts contain fat and can be confirmed with fat-suppressed images. Anyway, these pathologies do not usually appear with the clinical syndrome of PA.

## Role of imaging in prognosis, therapy and follow-up

In clinical practice, many endocrinologists and pituitary surgeons believe that patients presenting with ophthalmoplegia (typically III palsy) without visual field loss may safely be monitored conservatively—typically with spontaneous full recovery of the ophthalmoplegia after several weeks and very often with substantial shrinkage of the pituitary mass on the follow-up scan some months later.

The role of imaging, in particular of MRI, in the decision-making process for a proper therapy is still debated and probably needs more study. Some authors reported that MRI findings in patients with PA do not correlate with the severity of its clinical presentation, in terms of both visual loss and cranial nerve palsies [25, 45, 50], suggesting that the role of imaging might be limited. Despite this, in the series of Ayuk and colleagues, patients undergoing MRI were managed more safely with conservative treatment because of identification of areas of haemorrhage and infarction, which on repeated MRI scanning allows prediction of the evolution of PA [25]. Semple et al. recently described that patients with PA and MRI findings of pituitary infarction without signs of haemorrhage showed a less severe clinical presentation and a better outcome [14].

Although no study demonstrated that chiasm compression may represent the most relevant MRI feature, it is likely that this observation has a clinical impact in the decision-making process (Fig. 3).

**Fig. 7:**
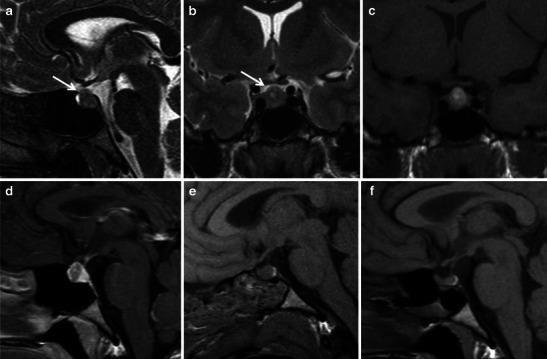
Pituitary apoplexy in a patient with pre-existing adenoma. Arrows on sagittal and coronal T2WI (**a**, **b**) as well as in coronal T1WI show an area of haemorrhage within the adenoma. Haemorrhagic components appear hyperintense on both T2WI and T1WI. In (**d**) sagittal contrast-enhanced T1WI showing peripheral enhancement. Despite the absence of chiasm compression and cavernous sinus involvement, this patient underwent surgical treatment with a transsphenoidal approach. Sagittal T1WI follow-up during surgery on intraoperative MRI (Brain Suite) (**e**) and late follow-up (3 months later, **f**) show good imaging results

For the above-mentioned reasons, even though the most important features for treatment decisions (surgical or conservative) are related to the severity of the clinical condition [59], MRI can offer relevant prognostic information (Figs. 3 and 7).

The recent UK guidelines for the management of PA indicate an urgent MRI in patients with suspected PA as IIIB evidence class, since more randomised clinical trials are required. If MRI is contraindicated, a dedicated pituitary multidetector CT is advisable [59].

**Fig. 8:**
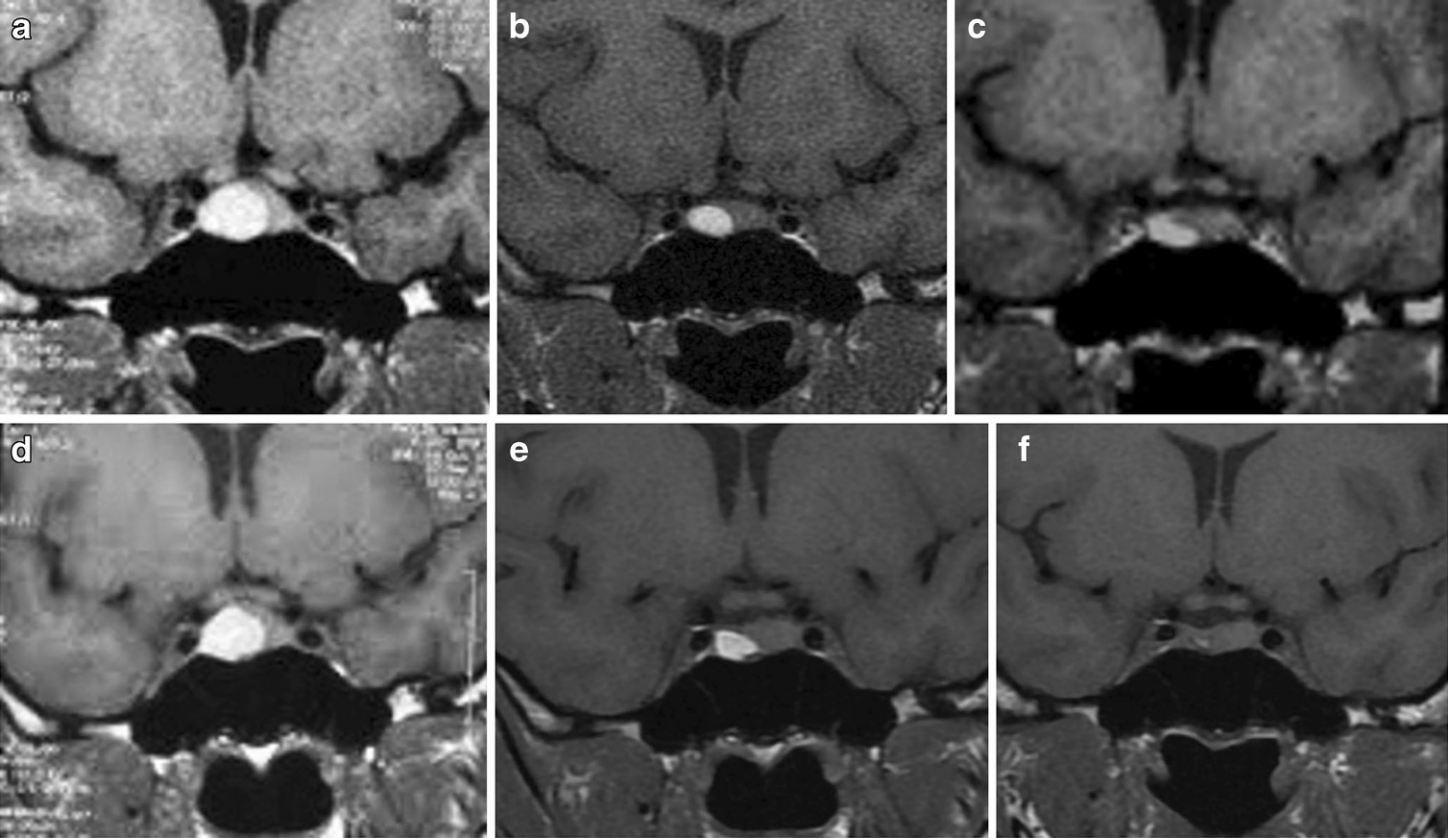
Series of coronal T1WI follow-up images in a patient with a history of pituitary apoplexy. The patient presented to us for observation in July 2008 with sudden headache (**a**). He underwent conservative treatment. Further MRI studies demonstrated progressive reduction of the haemorrhagic component over time (**b**, **c**). Because of a new sudden episode of headache, he underwent a new follow-up (**d**) that showed a new increase in the size of the T1WI hyperintensity, suggesting re-bleeding. Further follow-up showed a reduction of haemorrhage during the next 2 years

Radiological follow up with MRI in patients affected by PA is important. The pre-existing adenoma might grow after bleeding and eventually re-bleed (Fig. 8) [20]. In those patients treated conservatively, tumour growth after bleeding occurs within a wide range, as shown by different studies (from 6 to 90 %), but this indicates the need for clinical radiological follow-up 3–6 months after PA and every year for at least 5 years. Recurrent pituitary apoplexy has been documented in studies with both conservative and surgically therapeutic approaches, without a significant difference in the incidence between these groups [25, 60].

## Conclusions

PA is a severe clinical syndrome and imaging is important to rule out other pathologies showing similar symptoms.

Currently, CT is the most commonly used imaging modality during the acute onset of symptoms consistent with PA. Despite this, CT lacks sensitivity and MRI should always be performed if PA is suspected. MRI can evaluate pituitary infarct and haemorrhage, mass effect and differentiate PA from other pathologies such as a Rathke cleft cyst or craniopharingioma. Other MR techniques such as MRA, DWI and T2*-weighted GE images can increase its diagnostic capabilities.

Although more studies will be necessary to define the role of MRI in treatment choices and prognostic information, this modality is a fundamental step in the diagnosis of PA in the acute phase and is mandatory in the follow-up of these patients.
